# Interactions between gut commensal bacteria and polysaccharides derived from algae and legumes: identification of metabolites produced and pathways involved

**DOI:** 10.1016/j.crmicr.2026.100567

**Published:** 2026-02-10

**Authors:** Paul Biscarrat, Frederic Pepke, Clémence Defois-Fraysse, Aya Jeaidi, Christelle Hennequet-Antier, Olivier Rué, Florence Castelli, Céline Chollet, Cassandre Bedu-Ferrari, Jean-Yves Berthon, Cyril Chaudemanche, Assia Dreux-Zigha, Philippe Langella, Claire Cherbuy

**Affiliations:** a Micalis Institute, Institut National de Recherche pour l'Agriculture, l'Alimentation et l'Environnement (INRAE), AgroParisTech, Université Paris-Saclay, UMR1319, Jouy-en-Josas, France; b GreenCell, Biopôle Clermont-Limagne, 63360, Saint Beauzire, France; c Université Paris-Saclay, INRAE, MaIAGE, 78350, Jouy-en-Josas, France; d Université Paris-Saclay, INRAE, BioinfOmics, MIGALE bioinformatics facility, 78350, Jouy-en-Josas, France; e Université Paris-Saclay, CEA, INRAE, Département Médicaments et Technologies pour la Santé (MTS), MetaboHUB, Gif sur Yvette, F-91191, France; f General Mills - Häagen Dazs SNC, Arras, France

**Keywords:** Gut microbiome, Dietary fiber, Algae, Chickpea, Metabolomics, Transcriptomics

## Abstract

Diet is a key driver of gut microbiome functions, largely via microbial fermentation of dietary fibers. We investigated how 15 human gut commensals from Bacteroidota, Bacillota, and Actinomycetota metabolize structurally distinct poly-/oligosaccharides from algae (*Ulva lactuca, Saccharina latissima, Undaria pinnatifida*) and chickpeas (*Cicer arietinum*). In low-nutrient, carbon-defined cultures, we quantified growth (ΔOD), acidification (ΔpH), and short-chain fatty acids (SCFAs). Then, we conducted untargeted liquid chromatography–high-resolution mass spectrometry (LC-HRMS) metabolomics and RNA sequencing on eight representative strains. Chickpea raffinose-family oligosaccharides (RFOs) broadly stimulated growth, fermentation, and SCFA production across phyla, whereas algal polysaccharide use was restricted to specific Bacteroidota species. Metabolomics revealed phylum- and strain-resolved signatures and bioactive molecules beyond SCFAs, including tryptophan derivatives (for example, indolelactic acid), γ-aminobutyric acid (GABA), and micronutrient-related compounds (for example, riboflavin), whose abundance depended on both taxon and substrate. Transcriptomic analysis in the presence of raffinose indicated coordinated activation of carbohydrate-active enzymes (CAZymes), specialized transport systems (SusC/D, TonB, or ATP-binding cassette [ABC] transporters), and transcriptional regulators (for example, LacI), consistent with substrate-responsive carbohydrate gene clusters. Bacteroidota exhibited the largest CAZyme mobilization and transcriptional remodeling, while Bacillota and Actinomycetota showed targeted responses consistent with narrower substrate scopes.

**Conclusions:**

Fiber structure mechanistically links to selective microbial functions. Pulses-derived RFOs elicit broad, phylum-specific metabolic programs, and algae polysaccharides engage a limited set of Bacteroidota specialists. This integrative framework (growth, SCFAs, metabolomics, transcriptomics) refines how discrete fiber types can be matched to microbial capacities, informing precision-nutrition strategies that leverage sustainable fibers (pulses, algae) to promote health-relevant metabolites and targeted microbiome modulation.

## Highlights

• Chickpea oligosaccharides are broadly utilized by gut commensal bacteria.
• Algal polysaccharide use is limited to specific Bacteroidota species.
• Algae and chickpea fibers enhance short-chain faty acid production.
• Transcriptomics reveal coordinated genes for raffinose metabolism.

## Graphical abstract

**Figure:**
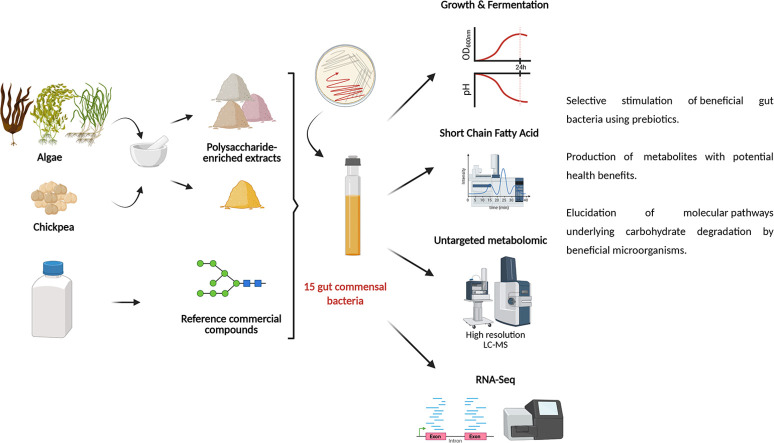

## 1 Introduction

The human gut hosts a diverse and abundant microbial community that plays a critical role in maintaining host health (VanEvery et al., 2023). While defining a "healthy" microbiome remains challenging (Joos et al., 2024; McBurney et al., 2019), a growing body of research highlights associations between the gut microbiota and the risk of chronic diseases as well as long-term health outcomes. Diet is one of the major modulators of microbiota composition, with plant-rich diets being consistently associated with favorable microbial profiles (Asnicar et al., 2021; Reynolds et al., 2019). Among dietary components, fibers are of particular interest, as they resist digestion by host enzymes and serve as substrates for microbial fermentation (Reynolds et al., 2019). Their degradation leads to the production of short-chain fatty acids (SCFAs) and other metabolites, which contribute to gut and systemic health (Deehan et al., 2022). However, fiber intake remains below recommended levels in many Western populations (Stephen et al., 2017), with consequences such as reduced microbial diversity (De Filippo et al., 2017), and even transgenerational microbiota depletion in both human and animal studies (Sonnenburg et al., 2016; Vangay et al., 2018). Numerous clinical and mechanistic studies have demonstrated how fiber-rich diets can beneficially shape microbiota composition and metabolic function (Holmes et al., 2022; Lancaster et al., 2022; Makki et al., 2018). While SCFAs such as acetate, propionate, and butyrate are well-recognized effectors, other fermentation products may also play important roles (Bedu-Ferrari et al., 2024). The initial microbial steps in fiber degradation involve carbohydrate-active enzymes (CAZymes) (Wardman et al., 2022), including glycoside hydrolases (GHs), which cleave glycosidic bonds. These enzymes are often encoded within polysaccharide utilization loci (PULs) (Kaoutari et al., 2013; Lombard et al., 2014; Martens et al., 2011), particularly in members of the Bacteroidota phylum. These capabilities are not evenly distributed across species and phyla, reflecting the strain-level variability in fiber utilization potential (Soverini et al., 2017). Dietary fibers themselves form a structurally diverse family, with variations in plant origin, sugar composition, linkage types, degree of polymerization, and molecular weight (Feng et al., 2025). These features influence their accessibility to microbial enzymes and thus determine which taxa are enriched. Recent reviews stress the importance of matching fiber structure to microbial metabolic capacity (Kok et al., 2023). While fibers from cereals, fruits, and vegetables are well studied, less is known about those from legumes and algae, two underexplored yet sustainable sources of dietary carbohydrates. Raffinose family oligosaccharides (RFOs) from legumes and sulfated polysaccharides from algae (e.g., ulvan, laminarin, fucoidan) offer contrasting structural profiles that could help fine-tune microbiota composition (Biscarrat et al., 2024; Diaz et al., 2023).

**Fig. 1:**
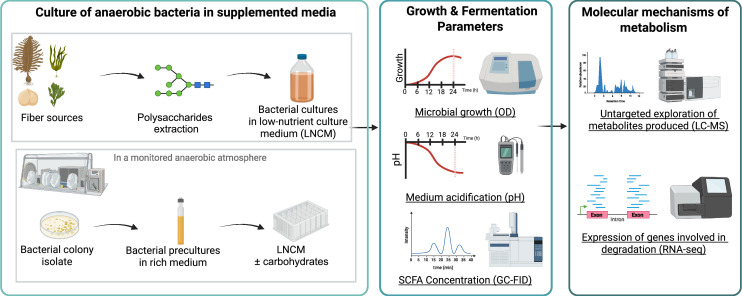
Experimental strategy for assessing the metabolic responses of anaerobic bacteria to polysaccharides. The study was conducted in two main phases. First, the impact of dietary fibers on the growth and fermentation activity of 15 representative gut bacteria was evaluated using fiber-enriched extracts from algae and chickpeas, as well as corresponding purified carbohydrates. Bacterial responses were assessed in terms of growth, acidification, and production of SCFAs. In a second phase, a deeper molecular characterization was carried out on a subset of eight strains from three phyla, focusing on their metabolic output and gene expression in response to selected fibers. This integrative approach allowed the identification of substrate-specific metabolic profiles and transcriptional signatures, offering insights into the functional specialization of gut commensals in fiber degradation.

Here, we investigate the interactions between 15 gut commensal bacteria and poly-/oligosaccharides derived from algae and chickpeas, using in vitro culture assays, untargeted metabolomics, and transcriptomic profiling. Our study aims to identify phylum-specific fermentation patterns, bioactive metabolite production, and molecular pathways involved in fiber utilization (Fig. 1). This work contributes to a deeper understanding of how fiber structure drives microbial metabolic responses and supports the development of targeted dietary strategies for microbiome modulation.

## 2 Materials and method

### 2.1 Extraction of poly/oligosaccharides from algae and pulses

Three types of algae (*Ulva lactuca, Saccharina latissima, and Undaria pinnatifida*) and one pulse (*Cicer arietinum*) were used to obtain polysaccharide preparations enriched in ulvan, laminarin, fucoidan, and RFO, respectively. Poly/oligosaccharide-enriched extracts from *Ulva lactuca, Saccharina latissima, Undaria pinnatifida* and *Cicer arietinum* were supplied by our partner GREENCELL (Biopôle Clermont-Limagne, Saint-Beauzire, France) according to the protocol described in (Uriot et al., 2025). Briefly, dried algae flakes underwent an acid hydrolysis followed by ultrafiltration and precipitation with 96% ethanol overnight. The precipitate was then dried in an oven to obtain the polysaccharide-enriched extracts. The *Cicer arietinum* chickpeas were crushed and underwent ethanolic extraction followed by a 5 kDa ultrafiltration. The permeate was then concentrated and freeze-dried to obtain the RFO extract (RFO MW 0.5–0.8 kDa). Characterization of each extract is provided in SupTable S1. Pure commercial compounds were used for comparison with the extracted compounds: raffinose (Sigma, R-7630), stachyose (MP Biomedicals, 153,946), and laminarin from *Laminaria digitata* (Sigma, L9634). Additionally, powdered long-chain inulin derived from agave was included in this study (Bedu-Ferrari et al., 2024).

### 2.2 Bacterial strains and inoculum cultures

A panel of 15 commensal gut bacteria, representing key members of the human gut microbiota, was selected to cover phylogenetic diversity across the Bacteroidota, Bacillota, and Actinomycetota phyla. The panel included: *Bacteroides intestinalis, Bacteroides xylanisolvens, Bacteroides thetaiotaomicron, Bacteroides fragilis, Bacteroides uniformis, Faecalibacterium duncaniae, Subdoligranulum variabile, Butyricicoccus pullicaecorum, Roseburia intestinalis, Agathobacter rectalis, Blautia hansenii, Anaerostipes caccae, Anaerobutyricum hallii, Bifidobacterium adolescentis* and *Bifidobacterium catenulatum* (See SupTable S2 for taxonomy and strain information). Bacterial strains were obtained from the Deutsche Sammlung von Mikroorganismen und Zellkulturen and the American Type Culture Collection. Genomic data, either draft or complete, were retrieved from the National Center for Biotechnology Information database (https://www.ncbi.nlm.nih.gov). Bacteria were isolated on supplemented BHI agar at 37°C within an anaerobic chamber containing a 90% N2, 5% CO2, and 5% H2 atmosphere (Coy Lab Products, USA). A single colony was cultured at 37°C for 24 h in supplemented BHI broth before being used as an inoculum culture diluted to an OD600 of 1 (Ultrapec 10 Cell Density Meter, Biochrom Ltd., UK). The counts of colony-forming units (CFU) were estimated to be in the range of 1 × 10^7 to 1 × 10^8 CFU/mL.

### 2.3 Culture conditions

Experimental culture conditions were carried out in low nutrient culture medium (LNCM), as previously described in (Bedu-Ferrari et al., 2024), where the sole carbon source was the one being investigated. All experiments were conducted under anaerobic conditions. LNCM was supplemented with algal extracts, chickpea extracts, inulin, commercially sourced pure compounds (raffinose, stachyose and laminarin), or glucose at a concentration of 0.5% (w/v). All carbon substrates were sterilized by 0.22 µm filtration. The reconstituted culture media were allowed to equilibrate for at least 48 h in the anaerobic chamber before being inoculated with 2% of the inoculum culture. Negative controls were systematically included in each experiment and consisted of the non-inoculated corresponding media and the inoculated basal LNCM without any added carbohydrates.

### 2.4 OD_600nm_ and pH measurements

Cultures were performed in duplicate in 2-mL 96-well plates, which were securely sealed with a sterile membrane (Thermo Scientific, USA) to prevent evaporation. The cultures were maintained under anaerobic conditions for 24 h at 37°C. After homogenizing the samples, portions of each monoculture were transferred to new 96-well plates (Corning, USA) for optical density (OD) at 600 nm (Tecan Infinite 200 Pro Plate Reader, Austria) and pH (Mettler Toledo, Switzerland) measurements. To assess bacterial growth and the extent of carbohydrate utilization, we calculated OD and pH variations between T0 and T24 for each bacterial culture, where T0 is the value obtained just after the inoculation of the bacteria. The non-inoculated LNCM was used as a blank.

### 2.5 SCFA analysis

SCFA content was measured on the deproteinized culture supernatant using gas chromatography (GC) equipped with a flame ionization detection (FID) (Agilent 7890 GC System) as described in (Bedu-Ferrari et al., 2024).

### 2.6 Metabolomics

After 24 h of incubation, samples were centrifuged at 12,000 g for 15 min at 4°C, and the supernatants were immediately stored at −80°C until metabolomic analyses were performed using Liquid chromatography coupled with high resolution mass spectrometry (LC-HRMS) (Bedu-Ferrari et al., 2024). Five to six replicates of each condition were carried out. After peak detection and integration, metabolite annotation was accomplished using an in-house chemical database by matching precise measured retention time (RT) and mass-to-charge ratio (*m/z*) to those of over 1000 pure authentic standards evaluated under identical conditions. Each annotated metabolites were categorized into classes of metabolites as follows: A and AE: Nucleosides, Nucleotides, and Analogues; E and AB: Organic Acids and Derivatives; V: Organic Acids and Derivatives/Lipids; G, J and AK: Amino Acids, Peptides, and Analogues ; D: Aliphatic Acyclic Compounds; O and AJ: Aliphatic Heteromonocyclic or Homomonocyclic Compounds; L and U: Carbohydrates and Carbohydrate Conjugates; N: Lipids/Organic Acids and Derivatives; X: Lipids and Lipids/Lipids; P and F: Aromatic Heteropolycyclic Compounds; K, AH and C: Aromatic Homomonocyclic Compounds; AF: Organophosphorus Compounds; I: NA. The identity of unannotated metabolites was also investigated by querying public databases (KEGG, HMDB, Metlin).

### 2.7 Transcriptome sequencing and analysis

The bacterial cultures were collected 12 h after inoculation of the culture media, which corresponds to the mid-exponential growth phase for all bacteria, except for *B.adolescentis*, which is between the mid- and late-exponential phase (data not shown). This timing allows for working at an active stage of substrate assimilation in the medium. Cultures were centrifuged at 12,000 × *g* for 15 min at 4°C, and pellets were immediately frozen at −80°C until RNA extraction and transcriptomic sequencing was performed using Illumina NextSeq500 (Bedu-Ferrari et al., 2024). Three replicates of each condition per bacteria were carried out.

### 2.8 Statistical analysis

All analyses were performed using R Studio v4.0.0. For OD600 and pH measurements, as well as SCFA quantifications, non-parametric Wilcoxon rank-sum tests were used to compare conditions with specific carbon sources to the corresponding basal control (LNCM without supplementation). Classification of metabolomic data was conducted using sPLS-DA, implemented in mixOmics v6.22.0 (Rohart et al., 2017). To identify significant features that differed between bacterial species for a given carbon source, univariate non-parametric tests were performed comparing inoculated LNCM to the corresponding non-inoculated medium (Wilcoxon tests with Benjamini–Hochberg (BH) adjustment to control for multiple testing, significance defined as Padj < 0.05).

Transcriptomic differential expression analyses were conducted using edgeR v3.38.4, employing generalized linear models and likelihood ratio tests (McCarthy et al., 2012). Genes were considered significantly differentially expressed between LNCM-raffinose to the reference condition, LNCM-glucose for Padj < 0.05 (Wilcoxon tests with BH adjustment). To identify genomic regions involved in carbohydrate metabolism, CAZyme-encoding genes were annotated using the dbCAN2 meta server, based on concordant results from HMMER, DIAMOND, and Hotpep searches against the CAZy database (Zhang et al., 2018). Clusters of carbohydrate-related genes were then defined by the co-localization of CAZymes with functionally related genes (e.g., transporters, regulators). The differential expression data were then mapped onto these regions to identify clusters specifically upregulated in response to raffinose compared to glucose. These clusters were defined as Carbohydrate Gene Clusters (CGCs).

Heatmaps were generated using the Heatmap function in the ComplexHeatmap package (Gu, 2022). Hierarchical clustering was conducted with default parameters unless specified otherwise. For SCFA data, hierarchical clustering was based on the dendextend package using the hclust function with the "Ward. D2" and the distance matrix was obtained with the vegdist function of the 'vegan' package with the "Bray" method corresponding to the Bray-Curtis index (Oksanen et al., 2025). The description of hierarchical categories was performed using the FactoMineR v2.8 (Lê et al., 2008). For metabolomic data, heatmap annotations were added to indicate metabolites that were shared or not shared with other culture media, as well as the class to which each metabolite belonged. A second heatmap was generated to display the mean fold-change per row (i.e., per bacterium), along with a boxplot summarizing the mean fold-change for each cluster given from the main heatmap.

## 3 Results

### 3.1 Caracterisation of poly/oligosaccharide extracts from algae and chickpeas

Poly- and oligosaccharide-enriched extracts were obtained from three species of algae (*Ulva lactuca, Saccharina latissima, Undaria pinnatifida*) and from chickpeas (*Cicer arietinum*). We found that total sugar amounts accounted for 28 to 51% according to the extract (SupTable S1). The monomer composition is consistent with the expected poly- and oligosaccharides (SupTable S1). To further characterize bacterial metabolic responses, selected purified carbohydrates (raffinose, stachyose, laminarin) were also tested in parallel.

### 3.2 Chickpea oligosaccharides are efficiently utilized by the majority of the studied bacteria, while the use of algal polysaccharides is restricted to certain species of bacteroidota

**Fig. 2:**
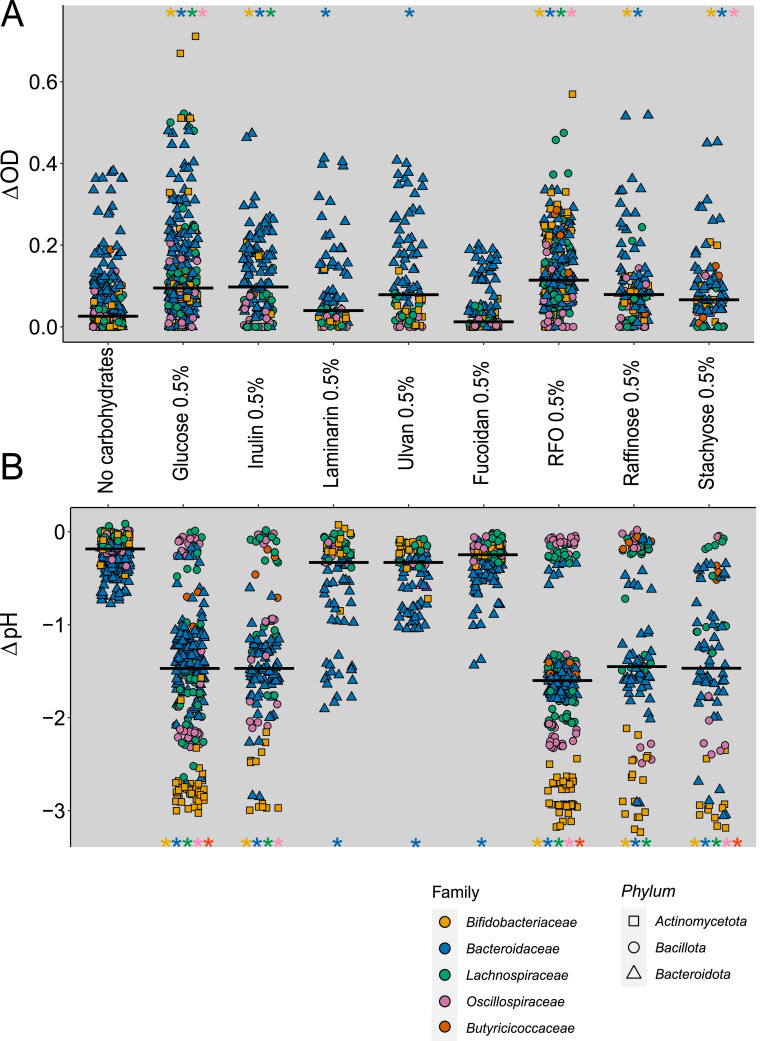
Evaluation of the growth of 15 intestinal commensal bacteria and the associated pH changes in the presence of fibers derived from algae and chickpeas. **(A)** Bacterial growth (ΔOD) and **(B)** medium acidification (ΔpH) after 24 h of culture with various carbohydrate sources. Conditions include a negative control (no carbohydrates), positive controls (glucose and inulin), fiber-enriched extracts from algae (laminarin, ulvan, fucoidan) and chickpeas (RFO), and purified oligosaccharides (raffinose, stachyose). Bacterial responses are grouped by family and phylum. For each bacterial family, values obtained under each carbohydrate condition were compared to the no-carbohydrate control using a Wilcoxon rank-sum test. Statistical significance is indicated on the plots by asterisks (*p*< 0.05), with asterisk colors matching the family color code shown in the legend. Results for individual strains are shown in Supplementary Figures S1 (Bacteroidota), S2 (Actinomycetota), and S3 (Bacillota).

We assessed the ability of 15 commensal gut bacteria to ferment extracts enriched in poly/oligosaccharides derived from algae and chickpeas, or their corresponding purified compounds, using LNCM as basal medium (Fig. 2 and SupFig. S1–S4). Bacterial growth was monitored via optical density (ΔOD) and fermentation via medium acidification (ΔpH) after 24 h. Negative controls (no carbon source) and two positive controls (glucose and inulin) were included. All Bacteroidota, Actinomycetota, and most Bacillota species exhibited significant growth and acidification with glucose (Fig. 2). Similar results were observed with inulin, except for a subset of Bacillota strains. Under negative control conditions, only Bacteroidota species showed detectable fermentation.

Algal polysaccharides were utilized exclusively by Bacteroidota (Fig. 2). Among them, *B. xylanisolvens* and *B. thetaiotaomicron* exhibited growth on ulvan, while *B. uniformis* grew on both laminarin and fucoidan (SupFig. S1, S2, S3). Commercial laminarin confirmed the observations from the algal extracts (SupFig. S4).

In contrast, the RFO extract from chickpeas supported fermentation in all bacteria except *F. duncaniae* and *B. hansenii*. The activity profiles observed with purified raffinose and stachyose were generally consistent with those obtained using the RFO extract for Bacteroidota, Actinomycetota, and several Bacillota strains. Some discrepancies were noted, especially in strains that showed fermentation with the RFO extract but not with pure raffinose or stachyose. This may be linked to compositional differences between extracts and purified compounds, as sucrose was detected in the chickpea extract (data not shown).

### 3.3 Dietary fibers from algae and chickpeas promote the production of SCFAs

**Fig. 3:**
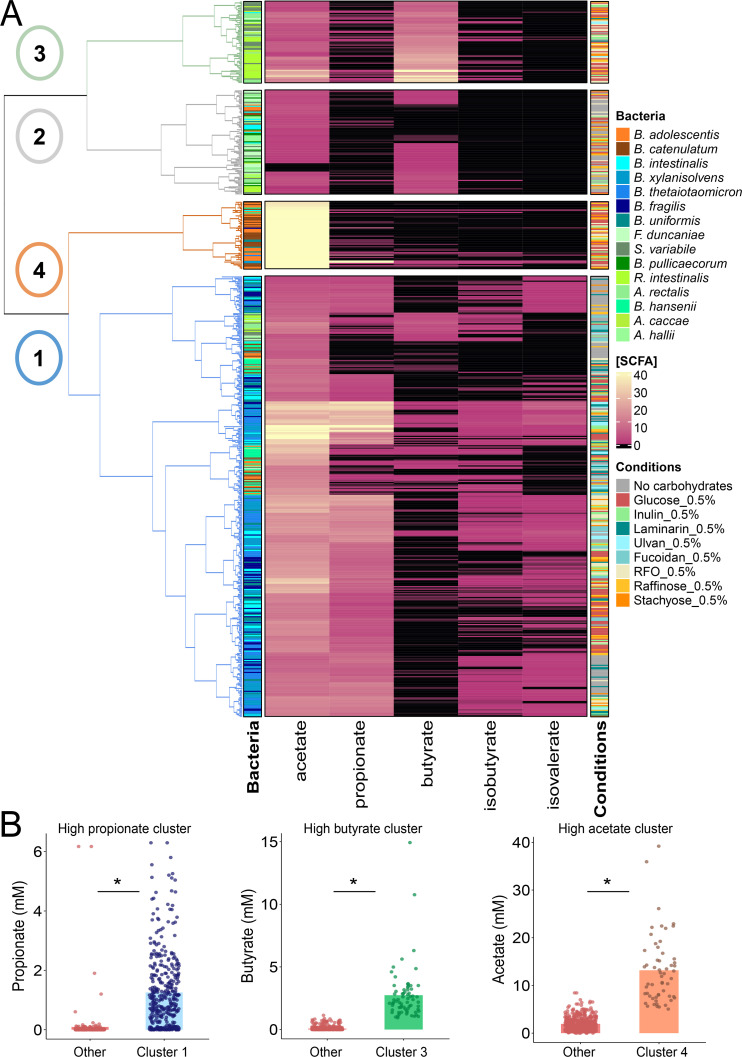
Classification of carbohydrate fermentation profiles of bacteria based on SCFA production. **(A)** Hierarchical clustering of bacterial monocultures based on SCFA production, revealing functional capacities for carbohydrate fermentation. Conditions include a negative control (no carbohydrates), positive controls (glucose and inulin), fiber-enriched extracts from algae (laminarin, ulvan, fucoidan), a chickpea-derived oligosaccharide extract (RFO), and purified oligosaccharides (raffinose, stachyose). SCFA concentrations (mM) are indicated by color intensity. Four distinct fermentation clusters are identified, with characteristics detailed in SupTable S3. **(B)** Comparison of the most abundant SCFA concentrations across the four clusters. Statistical differences were assessed using Wilcoxon tests (*P< 0.05).

SCFAs, including acetate, propionate, butyrate, isobutyrate, and isovalerate, were quantified in culture supernatants after 24 h incubation (Fig. 3). Hierarchical clustering based on SCFA profiles identified four distinct groups of bacterial responses.

One cluster showed minimal SCFA production and included negative controls and non-growing bacteria. A second cluster, mainly comprising Bacillota from Lachnospiraceae, Butyricicoccaceae, and Oscillospiraceae, produced high levels of butyrate, particularly in the presence of glucose, inulin, and chickpea RFO (mean: 2.74 ± 2.11 mM). A third cluster, composed of Bifidobacteria, was characterized by high acetate levels, especially under glucose and RFO conditions (mean: 13.18 ± 7.10 mM). The fourth cluster, composed predominantly of Bacteroidota, showed propionate production (mean: 1.26 ± 1.27 mM) and broader substrate utilization, including algae-derived polysaccharides. A Chi-square test revealed a significant association between SCFA production clusters and bacterial phylogeny (SupTable S3).

Based on these fermentation responses, a subset of eight bacterial strains was selected for further metabolomic and transcriptomic analysis. These included three Bacteroidota, three Bacillota, and two Actinomycetota strains, chosen based on their metabolic activity in the presence of carbohydrates. Because pulse-derived oligosaccharides are the most effective at stimulating the growth and metabolic activity of bacteria of interest, we focused on fibers extracted from chickpeas and excluded those derived from algae in the subsequent steps.

### 3.4 Metabolomic profiling reveals distinct metabolic responses to chickpea-derived oligosaccharides across different gut bacteria

**Fig. 4:**
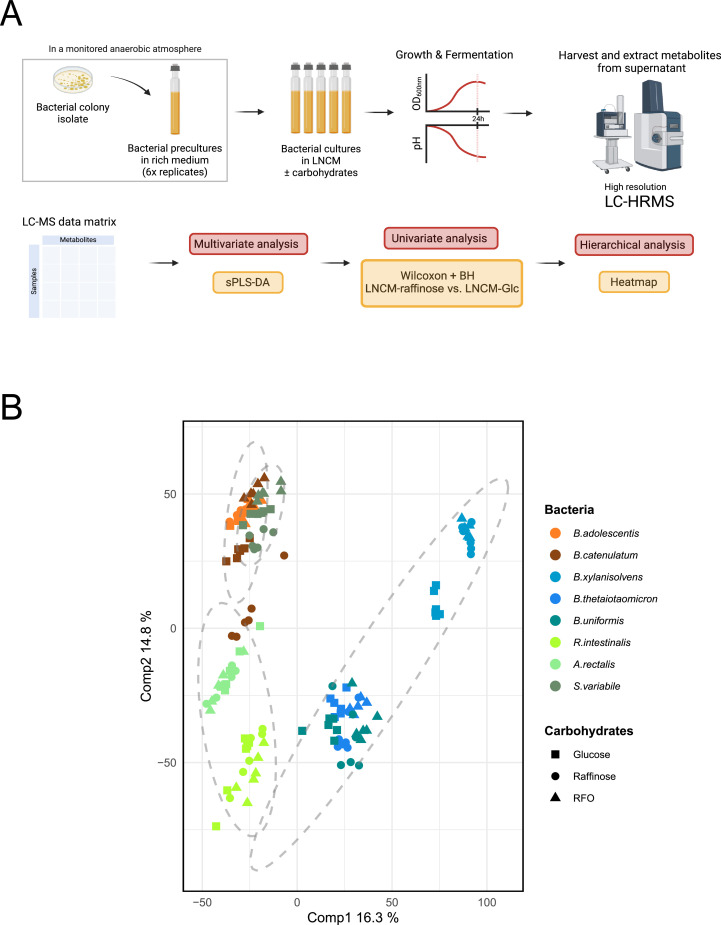
Metabolomic profiles of commensal bacteria based on culture conditions and carbon sources. **(A)** Schematic of the experimental strategy for untargeted metabolomic data analysis. The metabolomic study was performed on bacterial supernatants after 24 h of culture in low-nutrient culture media (LNCM), each supplemented or not with different carbon sources. Each condition was performed in six replicates, along with five replicates of the initial non-inoculated LNCM **(B)** sPLS-DA plot illustrating the clustering of metabolomic profiles according to bacterial phyla and culture conditions with glucose, raffinose, or RFO. Distinct metabolic signatures are observed for each phylum. Results for individual strains are shown in SupFig. S5.

Non-targeted, LC-HRMS metabolomics were employed to enhance our understanding of the metabolic responses of the eight bacteria when exposed to different carbohydrates (Fig. 4A). In total, 185 metabolites were confidently annotated using an in-house database, while 3838 compounds, not identified by the in-house database, were matched and annotated using public databases. Additionally, 5855 features remained unidentified. Sparse partial least squares discriminant analysis (sPLS-DA) reveals that metabolomic profiles predominantly cluster according to bacterial phyla (Fig. 4B). Specifically, the metabolic profile of Bacteroidota is distinct from those of Actinomycetota and Bacillota, regardless of culture conditions (Fig. 4B).

Within each phylum, we also observed distinct metabolomic profiles for most individual strains. The metabolomic profile of *S. variabile* (which belongs to Oscillospiraceae) was distinct from those of *A. rectalis* and *R. intestinalis* (which belong to Lachnospiraceae). In fact, *S. variabile* clustered more closely with the Actinomycetota. *B. thetaiotaomicron* and *B. uniformis* exhibited overlapping metabolomic profile, which differed from that of *B. xylanisolvens.* (Fig. 4B). The addition of carbohydrates has a specific impact on the metabolomic profile of each strain (SupFig. S5). For strains belonging to Actinomycetota (SupFigs. S5A and S5B) and Bacillota (SupFigs. S5C, S5D and S5E), we observed significant divergence in their metabolomic profiles when exposed to different carbon sources compared to the basal LNCM. For Bacteroidota (SupFigs. S5F, S5G and S5H), the data demonstrate metabolic activity in the basal medium, without carbohydrates, confirming their broad metabolic capacity. The addition of carbohydrates induces a metabolomic shift, which is notably more pronounced with oligosaccharides (Raffinose and RFO) than with simple sugars (glucose).

Taken together, these results indicate that the production of metabolites can be influenced by the nature of the substrates (carbohydrates) in the culture medium, in a phylum-dependent manner.

### 3.5 Metabolomic analyses reveal the production of potentially beneficial metabolites within bacterial communities

**Fig. 5:**
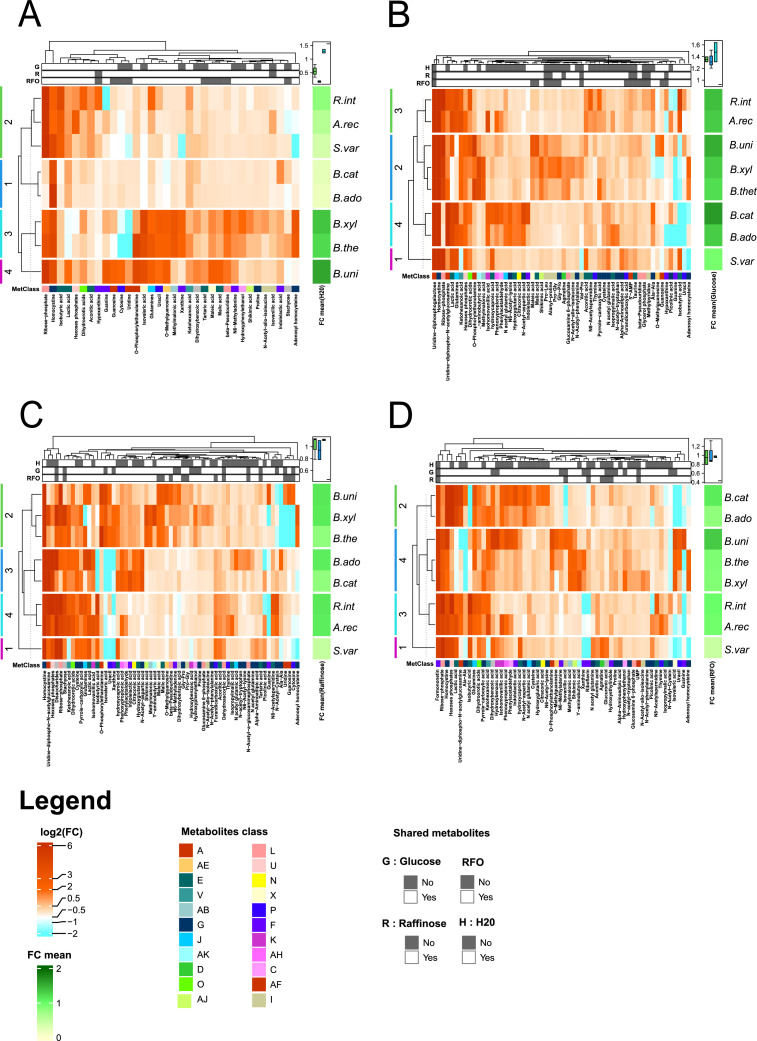
Bacterial metabolites whose abundance is modified compared to the non-inoculated media. Heatmaps of differentially abundant metabolites following 24 h of bacterial incubation under four conditions: without carbohydrate **(A)**, with glucose **(B)**, with raffinose **(C)**, or with chickpea-derived RFO **(D)**. The bacteria were named as follows: B.xyl: Bacteroides xylanisolvens; B.the: Bacteroides thetaiotaomicron; B.uni: Bacteroides uniformis; S.var: Subdoligranulum variabile; R.int: Roseburia intestinalis; A.rec: Agathobacter rectalis; B.ado: Bifidobacterium adolescentis; B.cat: Bifidobacterium catenulatum. Metabolites shown in the heatmap are those that (i) are annotated with a high level of confidence (confirmed by reference standards) and (ii) significantly differ between inoculated and the corresponding non-inoculated media, as identified by a Wilcoxon test with BH correction (Padj < 0.05) and a fold change ≥ 2. . (See also SupFig. S6 for metabolites annotated only based on public database matching, and SupTable S4 for the complete list of metabolites, annotation confidence levels, and associated statistics). The colored lines to the left of the heatmap identify clusters, with the associated dendrogram. The top annotation bar indicates whether a metabolite is shared across conditions, and the bottom annotation shows metabolite classes (see Materials and Methods for class correspondence). The right-hand heatmap and boxplot summarize the mean fold change per strain and per cluster, respectively.

We then focused on the metabolites that differ between bacterial strains and culture conditions. Specifically, we examined the metabolites whose identities were confirmed using our in-house database (Fig. 5), while metabolites identified solely through queries to public databases are presented in SupFig. S6. Heatmaps illustrate metabolites whose abundance significantly changes after 24 h of incubation in the presence of bacteria, compared to the non-inoculated LNCM.

The number of differentially abundant metabolites increased in the presence of carbohydrates: 34 for non-supplemented LNCM, vs. 58, 63, and 56 for glucose, raffinose, and RFO, respectively (See Fig. 5A for non-supplemented LNCM, Fig. 5B for LNCM-glucose, Fig. 5C for LNCM-raffinose, Fig. 5D for LNCM-RFO and SupTable S4 for the list of metabolites). The metabolites produced in the non-supplemented LNCM are mainly due to Bacteroidota metabolism. Interestingly, a large number of metabolites produced in the presence of carbohydrates are not shared with the basal condition (Fig. 5).

Regardless of the culture conditions, the metabolomic profile clusters by phyla or families. A similar pattern is observed for metabolites annotated with public database (SupFig. S6). Thus, we found distinct metabolic signatures with metabolites specific to bacterial groups. Maleic acid/fumaric acid (named maleic acid on the heatmap), malic acid and methylmalonic acid/succinic acid (named methylmalonic acid on the heatmap) production are specific to Bacteroidota and are produced whatever the culture conditions, while isobutyric acid is a signature of Bacillota.

Our findings revealed enrichment in metabolites associated with glycolysis, pentose phosphate pathway, and pyruvate catabolism in presence of carbohydrates. Hexose phosphates (corresponding, for example, to Glucose1-phosphate; Mannose1-phosphate; Mannose6-phosphate) increase in all conditions with carbohydrate and not in basal LNCM (Fig. 5). Similarly, uridine diphosphogalactose strongly increase in presence of glucose. Some of the bacteria group-specific metabolites are modulated by carbohydrate: this is the case with lactic acid, whose abundance is increased in the presence of sugars with Actinomycetota and Bacillota*.*

Of all the metabolites produced, we identified potentially beneficial metabolites. This is the case of: indolelactic acid, which is primarily produced from Actinomycetota*,* such as *B. catenulatum;* of *t*he inhibitory neurotransmitter, γ-aminobutyric acid (GABA); and of shikimic acid, which are increased in the presence of complex carbohydrates for Bacteroidota, primarily with *B. xylanisolvens*. Also, some metabolites related to vitamins were slightly but significantly increased: this is the case of riboflavin (vitamin B2) which is specifically produced from RFO in our study, and of dehydroascorbic acid, an oxidized form of ascorbic acid, which is produced by the Bacillota species in the presence of raffinose (Fig. 5).

### 3.6 Transcriptomic analysis and CAZymes expression in bacteria cultured with raffinose

**Fig. 6:**
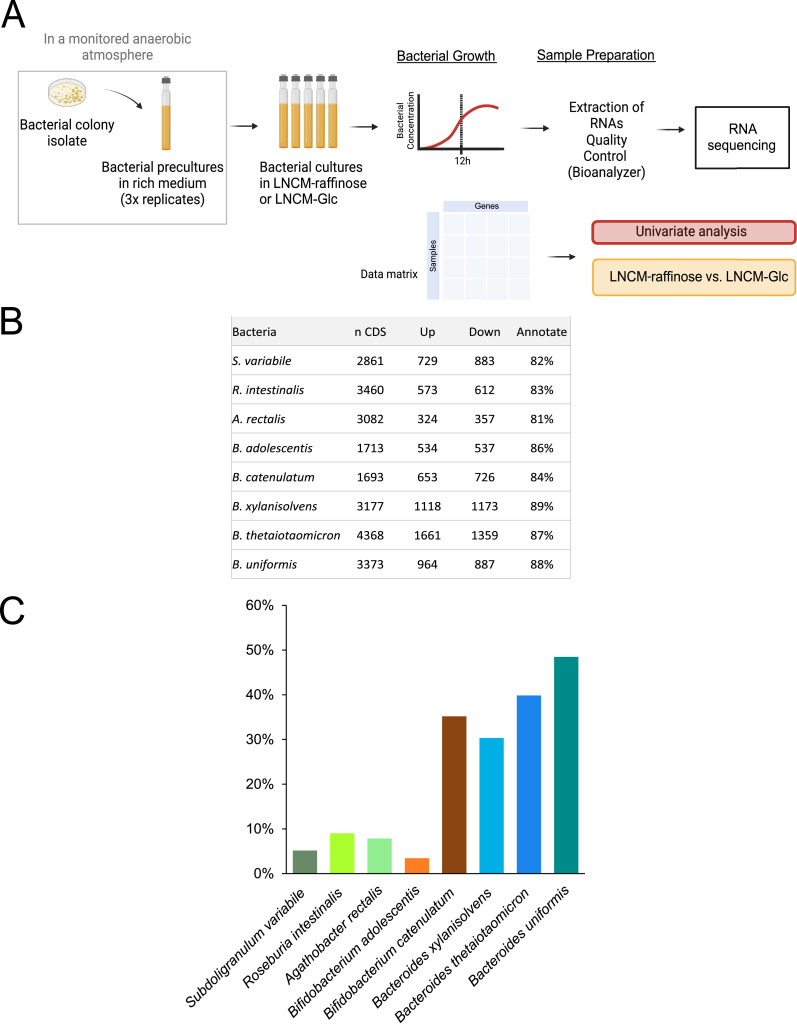
Transcriptomic analysis of the response to raffinose in selected gut bacteria. **(A)** Overview of the transcriptomic experiment performed on eight commensal bacterial species from Bacillota, Actinomycetota, and Bacteroidota, cultured with raffinose or glucose for 12 h. **(B)** Number of genes up- or downregulated in response to raffinose compared to glucose, along with the percentage of annotated coding sequences (CDS) per genome. **(C)** The percentage of CAZymes that are responded upregulated (i.e., differentially expressed) under raffinose conditions among the total number of CAZymes predicted by the genome.

In this phase of the study, we focused on raffinose, as a model oligosaccharide found in pulses. RNA sequencing was performed on the eight selected bacterial strains cultured with raffinose or glucose (as a reference condition for transcriptomic analysis) for 12 h, allowing for working at an active stage of the bacterial metabolic activity (Fig. 6A). The gene expression analysis revealed a diverse range of transcriptional responses to raffinose across the eight bacterial strains, with significant variation in the number of upregulated and downregulated genes, compared to the glucose condition (Fig. 6B). Of the Bacteroidota, *B. thetaiotaomicron*, which is considered one of the key polysaccharide breakdown member in the intestinal microbiota, exhibited the most pronounced response, with 1661 genes upregulated and 1359 downregulated when compared to glucose (Fig. 6B). In contrast, *A. rectalis* exhibited a more limited transcriptional response, with 324 genes upregulated and 357 downregulated. *S. variabile* and *R. intestinalis*, and Actinomycetota displayed intermediate levels of gene modulation, versus the reference condition. High annotation rates (85–90%) across strains enabled a detailed interpretation of these transcriptional changes.

Fig. 6C further illustrates the percentage of CAZymes that are responded upregulated (i.e., differentially expressed) under raffinose conditions among the total number of CAZymes predicted by the genome . *B. uniformis* and *B. thetaiotaomicron* display the highest percentages, highlighting their ability to produce enzymes involved in polysaccharide breakdown while *S. variabile* and *R. intestinalis* show lower levels of CAZyme upregulation under the same conditions. Interestingly, *B. catelunatum* also mobilizes around 30% of the CAZymes repertoire in the presence of raffinose. These findings emphasize the role of raffinose in stimulating the expression of glycan-degrading genes, primarily among Bacteroidota species, but also in some Actinomycetota species, compared to glucose, a simple sugar.

### 3.7 Exploration of CAZyme genomic environment and identification of potential carbohydrate gene clusters

Next, we focused on genes in the vicinity of the most highly expressed CAZymes, specifically those ranked within the top 100 expressed genes. This allows to identify co-expressed genes and potential CGCs, which include, in addition to CAZymes, transporters, signal transduction proteins, and transcription factors, to form an integrated system for raffinose processing and transport (Deehan et al., 2022) (See SupTable S5 for details of the genes).

**Fig. 7:**
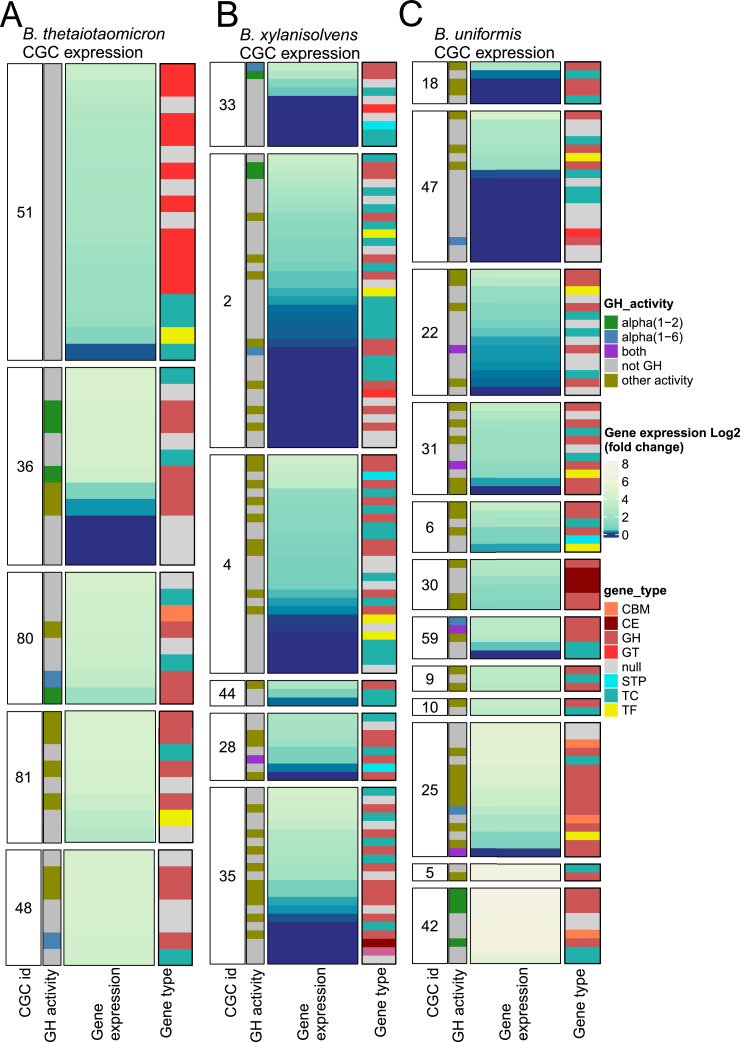
Analysis of the genomic context of highly expressed CAZymes in Bacteroidota and identification of co-expressed genes. Variation of gene expression (log₂ fold change) of carbohydrate-active enzymes (CAZymes) and associated genes in presence of raffinose versus glucose in Bacteroides thetaiotaomicron **(A)**, B. xylanisolvens **(B)**, and B. uniformis **(C)**. Each panel represent genes within potential Carbohydrate Gene Clusters (CGCs), highlighting CAZymes targeting α-1,2 and α-1,6 linkages, along with co-expressed transporters, regulators, and other functionally relevant proteins. Categories include GH (glycoside hydrolases), GT (glycosyltransferases), CE (carbohydrate esterases), CBM (carbohydrate-binding modules), TF (transcription factors), TC (transporters), STP (signal transduction proteins), and hypothetical proteins (null).

Notably, in the Bacteroidota species, significant co-expression patterns were observed, particularly between glycoside hydrolases and transport genes such as TonB and SusD/SusC, suggesting a highly optimized system for raffinose breakdown (Fig. 7). For example, among the potential CGCs we identified, one CGC of *B. thetaiotaomicron* (id: 51) was enriched in glycosyltransferases involved in glycoconjugate synthesis (Fig. 7A), while those of *B. uniformis* (id: 25) and *B. xylanisolvens* (id: 35) exhibited a variety of glycoside hydrolases capable of targeting different carbohydrates (Fig. 7B, C). Regulatory elements such as LytR, HCTS, and sigma factors were also identified, suggesting precise control over gene expression in response to substrate availability (SupTable S5).

**Fig. 8:**
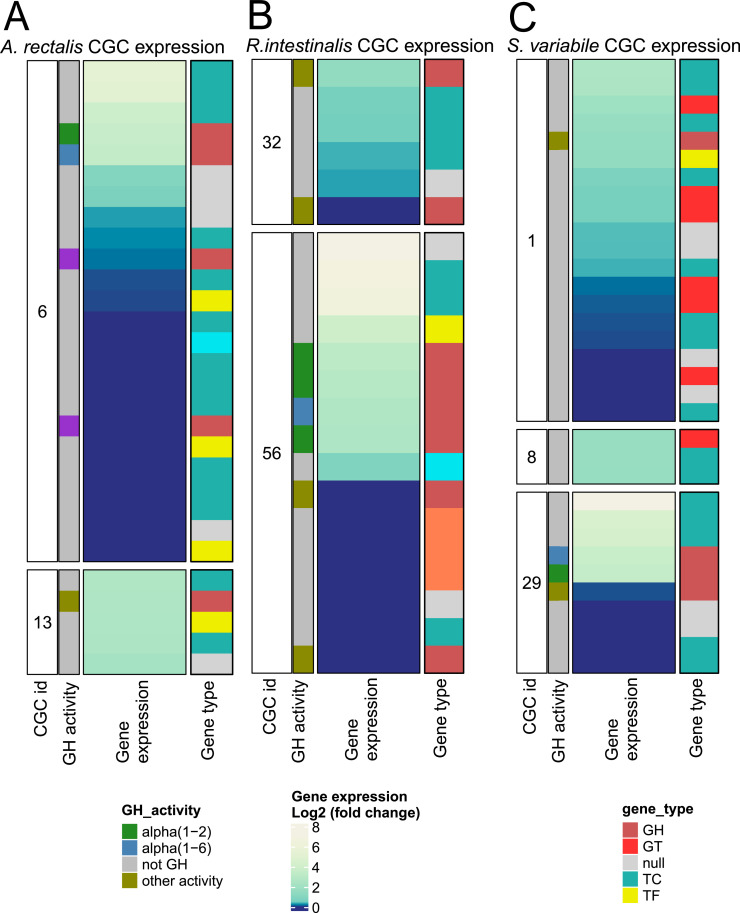
Genomic context of highly expressed CAZymes in Bacillota and identification of co-expressed genes. Variation of gene expression (log₂ fold change) of carbohydrate-active enzymes (CAZymes) and associated genes in presence of raffinose versus glucose in Agathobacter rectalis **(A)**, Roseburia intestinalis **(B)**, and Subdoligranulum variabile **(C)**. Each panel shows genes within potential Carbohydrate Gene Clusters (CGCs), including CAZymes targeting α-1,2 and α-1,6 linkages, along with co-expressed transporters, regulators, and other relevant proteins. Categories include GH (glycoside hydrolases), GT (glycosyltransferases), TF (transcription factors), TC (transporters), and hypothetical proteins (null).

Bacillota exhibited a distinctive configuration, with ABC-type transporters co-localized with CAZymes (Fig. 8), enhancing their capacity to digest host-derived hexoses and oligosaccharides. The presence of a LacI-type transcriptional regulator within these clusters suggests a coordinated control of carbohydrate metabolism, offering a potential competitive advantage in the gut’s nutrient-rich environment. This pattern is exemplified in *A. rectalis* (Fig. 8A), *R. intestinalis* (Fig. 8B), and *S. variabile* (Fig. 8C).

**Fig. 9:**
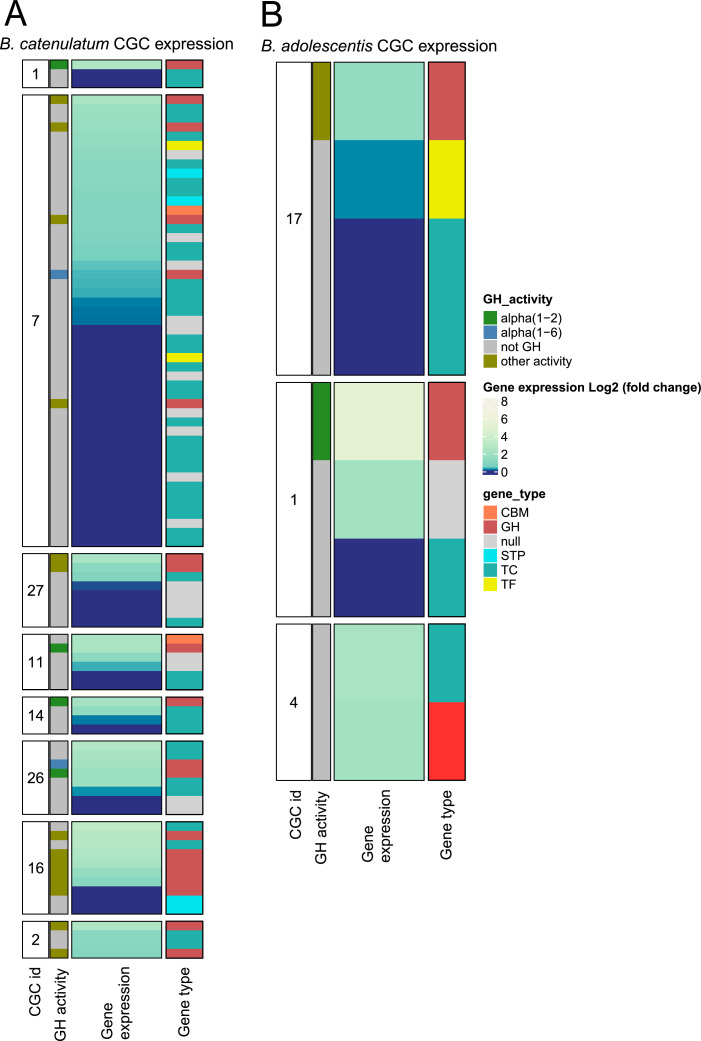
Genomic context of highly expressed CAZymes in Actinomycetota and identification of co-expressed genes. Variation of gene expression (log₂ fold change) of carbohydrate-active enzymes (CAZymes) and associated genes in presence of raffinose versus glucose in Bifidobacterium catenulatum **(A)** and Bifidobacterium adolescentis **(B)**. Each panel shows genes within potential Carbohydrate Gene Clusters (CGCs), including CAZymes targeting α-1,2 and α-1,6 linkages, along with co-expressed transporters, regulators, and other relevant proteins. Gene categories include genes encoding GH (glycoside hydrolases), CBM (carbohydrate-binding modules), TF (transcription factors), TC (transporters), STP (signal transduction proteins), and hypothetical proteins (null).

In Actinomycetota, similar genomic configurations were observed, particularly in *B. catenulatum*, which contained ABC-type transporters and LacI-type transcription factors, indicating an efficient strategy for nutrient uptake and metabolic flexibility (Fig. 9). This configuration, commonly found in the Bifidobacterium species, supports a versatile framework for carbohydrate metabolism, as demonstrated in *B. catenulatum* (Fig. 9A) and *B. adolescentis* (Fig. 9B).

Overall, despite differences in gene organization and metabolic strategies across Bacteroidota, Actinomycetota, and Bacillota, a common pattern to raffinose utilization emerged. This strategy involves the breakdown of raffinose by CAZymes, cellular import via specialized transporters, and regulation by transcription factors, emphasizing the adaptive and regulated nature of carbohydrate metabolism that contributes to their ecological success in the gut.

## 4 Discussion

### 4.1 Characterization of dietary fibers from algae and chickpeas and their taxonomic-specific utilization by gut microbiota

The evaluation of the growth and fermentation activity of gut bacteria on various polysaccharides revealed marked differences in carbohydrate metabolism between bacterial groups. In particular, while some bacterial groups do not utilize algal polysaccharides, certain species of Bacteroidota are able to metabolize them efficiently, resulting in the production of associated SCFAs, mainly propionate. Bacteroidota stood out for their versatile capacity to utilize the widest range of fibers, which can be attributed to their extensive repertoire of CAZymes specialized in breaking down complex polysaccharides (Wardman et al., 2022). The polysaccharide structures derived from algae are complex and require enzymatic repertoires that are possessed by Bacteroidota but not by other members of the microbiota. These metabolic capacities contrast with that of Actinomycetota and Bacillota, whose fermentation abilities appear restricted to inulin and chickpea oligosaccharides, suggesting specialization for certain dietary carbohydrates (Cockburn and Koropatkin, 2016; Kaoutari et al., 2013; Pokusaeva et al., 2011). Overall, this data shows that dietary prebiotics interact differently with different gut commensal bacteria, contributing to associated distinct SCFA production, which is phyla driven, e.g., propionate production for Bacteroidota, acetate production for Actinomycetota, and butyrate production for Bacillota. However, for the algal- and chickpea-derived extracts, although we verified that they contained the expected complex carbohydrates, we cannot exclude the presence of other bioactive components that may also contribute to the observed effects. For this reason, corresponding commercial oligo- and polysaccharides were used in parallel, and metabolomic and transcriptomic analyses were performed using the purified commercial compounds.

### 4.2 Metabolomic diversity and adaptive strategies in response to dietary fibers

In addition to SCFA production, a range of other metabolites generated by carbohydrate-metabolizing bacteria was identified in our study. Our findings highlight the capacity of pulse-derived polysaccharides, raffinose and RFO-enriched chickpea extract, to stimulate diverse metabolic activities across gut bacteria and the extensive metabolic shifts induced by these fibers. This underscored the bacteria's active DNA replication and efficient utilization of sugars, revealing their high capacity to metabolise these carbohydrates for energy. Distinct metabolic signatures were revealed among the phyla. Bacteroidota displayed remarkable metabolic flexibility, producing bioactive metabolites such as GABA, an amino acid that functions as the primary inhibitory neurotransmitter, and riboflavin (vitamin B2) (Li et al., 2017; Otaru et al., 2021). Interestingly, the latter is specifically produced by *Bacteroidota* when exposed to RFO. Similarly, Actinomycetota, including *B. adolescentis* and *B. catenulatum*, generated tryptophan derivatives, such as indolelactic acid (Meng et al., 2020) with potential roles in immune modulation and intestinal barrier maintenance (Agus et al., 2018; Ehrlich et al., 2020). Bacillota exhibited distinct adaptive strategies, producing metabolites such as lactic acid and taurine, which are associated with microbial and host health (Qian et al., 2023). LC-HRMS metabolomics revealed only 185 metabolites annotated by an in-house database and 3838 metabolites annotated by public database, revealing the importance of continuing to identify metabolites. Characterizing these compounds, many of which could offer significant benefits to the host, remains a complex and ongoing challenge in research (Krautkramer et al., 2021). This study takes a step forward by linking fiber-specific bacterial metabolism to unique metabolic profiles, emphasizing the role of raffinose and RFO in shaping bacterial activity. Investigating these metabolic profiles further could enable the identification of biomarkers that capture the influence of dietary fibers with potential prebiotic properties (Han et al., 2021). Future research should focus on validating the functional roles of these metabolites *in vivo*, to establish their relevance for therapeutic strategies aimed at optimizing gut health.

### 4.3 Transcriptomic insights into glycan breakdown and adaptive gene expression in gut bacteria

Using mono-culture experiments, we have begun to discover and describe carbohydrate gene machinery in relation to substrate. The transcriptomic analysis of bacterial responses to raffinose revealed distinct gene expression profiles that reflect the metabolic adaptability of each bacterial group. Bacteroidota species, particularly *B. thetaiotaomicron* and *B. uniformis*, showed extensive upregulation of genes involved in glycan breakdown, underscoring their capacity to utilize raffinose as an energy source. This heightened expression of CAZymes, notably glycoside hydrolases, suggests that Bacteroidota possess a robust enzymatic machinery to break down complex polysaccharides, conferring on them a competitive advantage in fiber-rich environments (Grondin et al., 2017). In contrast, Bacillota and Actinomycetota exhibited a more selective gene expression response, indicating specialization for particular substrates and a more limited but targeted enzymatic setup (Smith et al., 2020). The co-localization of CAZymes with transport and regulatory genes in potential CGC highlights an integrated system for raffinose metabolism, where genes for substrate breakdown, import and regulation are coordinated to optimize resource utilization (Martens et al., 2008). For example, the co-expression of transporters (e.g., TonB, SusD/SusC) with CAZymes in Bacteroidota suggests an efficient system for importing and processing raffinose (Joglekar et al., 2018), while the presence of LacI-type regulators in Bacillota and Actinomycetota points to a tightly regulated metabolic response to substrate availability (Sheridan et al., 2016). Overall, these findings demonstrate that raffinose can selectively induce glycan-degrading pathways in gut bacteria, supporting their ecological roles in the gut microbiota and illustrating the adaptive regulation of carbohydrate metabolism across diverse bacterial groups.

### 4.4 Dietary fibers as targeted tools for microbiome-driven health benefits

Dietary fibers are invaluable for modulating the composition and functionality of the gut microbiota, with well-established benefits extending to both gastrointestinal and systemic health (Barber et al., 2020; Fu et al., 2022). Notably, both stachyose and *Ulva lactuca* oligosaccharides have been shown to improve metabolic outcomes-such as glucose homeostasis, adipose tissue remodeling, and neuroendocrine signaling-through microbiota-dependent mechanisms involving specific taxa and metabolites (Chen et al., 2025, 2022). By leveraging specific fiber structures that promote the production of beneficial metabolites, dietary interventions offer a promising avenue for microbiome-targeted health strategies (Hutkins et al., 2024). Despite clear evidence supporting the health benefits of fiber, intake levels remain consistently below recommended thresholds in industrialized nations, posing a significant public health challenge (Ioniță-Mîndrican et al., 2022). This gap highlights the urgency for effective, fiber-based nutritional interventions to address these deficiencies (Delzenne et al., 2020).

Personalized approaches to fiber supplementation could optimize health outcomes by taking into account individual variations in microbiota composition and functional capacity (Deehan et al., 2020). Such approaches could also cater to specific populations, including those with health conditions requiring modified fiber intake levels (Fiorindi et al., 2022). Sustainable sources of dietary fibers, such as pulses and algae, offer dual benefits: high nutritional value combined with a low environmental footprint (Ferreira et al., 2021; Wu et al., 2023). These attributes align with global dietary transitions toward more sustainable food systems, emphasizing the role of these fibers in modern nutrition strategies.

The selective modulation of microbial taxa and the associated production of functional metabolites form the basis for developing personalized, fiber-based interventions. By connecting gene expression to metabolic pathways, we show how fibers can trigger specific microbial responses, thus supporting the broader idea of targeted microbiome modulation (Li et al., 2022). Our Work is based on individual strains of the gut microbiome and future research should focuse on validation of these findings to confirm their impact within the complexity of the gut environment. Such studies will be critical in advancing dietary fibers as central components of adaptive and functional nutrition frameworks.

## 5 Conclusion

This study provides an integrative view of how structurally distinct dietary fibers-derived from algae and chickpeas-are differentially utilized by gut commensal bacteria. By combining growth assays, SCFA quantification, untargeted metabolomics, and transcriptomics, we reveal strain- and phylum-specific metabolic adaptations that underpin the microbial response to fibers. Notably, these responses are shaped by the taxonomic identity and enzymatic repertoire of each bacterial group, highlighting the functional diversity of the gut microbiota in carbohydrate metabolism. Our findings show that beyond SCFA production, fiber-specific exposure triggers the synthesis of bioactive metabolites and activates targeted gene expression programs, particularly in CGC encoding CAZymes, transporters, and regulators. These data provide molecular insights into how gut bacteria adapt to complex substrates such as raffinose or marine polysaccharides, and establish a mechanistic link between fiber structure and microbial function. This reductionist *in vitro* approach offers a valuable framework for identifying candidate fibers with prebiotic potential, which may support the development of tailored nutritional strategies. In particular, sustainable fiber sources like pulses and algae emerge as promising tools for microbiome modulation, with relevance for both individual and planetary health. Future studies should focus on validating these strain-specific responses in complex ecosystems and human interventions. A deeper understanding of the interplay between fiber structure, bacterial metabolism, and host physiology will be essential to advance precision nutrition strategies that harness the microbiome as a lever for health promotion.

## Funding source

This work was supported by the French PSPC (Projet Structurant Pour la Compétitivité) RestorBiome project (grant number DOS0099202/00).

## Data availability

All summary data analyzed in this study is included in supplementary files, and the data sets of OD, pH, and SCFA in this study are available from the corresponding author upon reasonable request. The RNA-seq data set used in this study can be found in NCBI, GSE237111.

## CRediT authorship contribution statement

**Paul Biscarrat:** Methodology, Investigation, Writing – original draft. **Frederic Pepke:** Methodology, Investigation. **Clémence Defois-Fraysse:** Resources. **Aya Jeaidi:** Investigation. **Christelle Hennequet-Antier:** Formal analysis. **Olivier Rué:** Formal analysis. **Florence Castelli:** Investigation. **Céline Chollet:** Investigation. **Cassandre Bedu-Ferrari:** Methodology. **Jean-Yves Berthon:** Resources. **Cyril Chaudemanche:** Funding acquisition. **Assia Dreux-Zigha:** Resources. **Philippe Langella:** Funding acquisition, Supervision. **Claire Cherbuy:** Funding acquisition, Supervision, Writing – review & editing.

## Declaration of competing interest

The authors declare the following financial interests/personal relationships which may be considered as potential competing interests: CCHE reports financial support was provided by BPI France. CDF, ADZ and JYB reports a relationship with Greencell that includes: employment. ADZ reports financial support was provided by Riom Limagne &Volcan and Région Auvergne-Rhône-Alpes. CCHA reports a relationship with General Mills - Häagen Dazs that includes: employment. The other authors declare that they have no known competing financial interests or personal relationships that could have appeared to influence the work reported in this paper.

## Appendix Supplementary materials

Image, application 1

Image, application 2

Image, application 3

Image, application 4
