# Supplementary material for: Interactions between gut commensal bacteria and polysaccharides derived from algae and legumes: identification of metabolites produced and pathways involved
Source: Curr Res Microb Sci. 2026 Feb 10;10:100567. doi: 10.1016/j.crmicr.2026.100567 (PMC12924908; doi:10.1016/j.crmicr.2026.100567)
Supplement: Supplementary file 1 [file mmc1.pdf]

Supplementary Figure S1 : Growth and fermentation activity of intestinal commensal bacteria of the *Bacteroidota* phylum, reported individually, in the presence of fibers derived from algae and chickpeas.

Bacterial growth and fermentation activity were assessed by measuring optical density (OD600) and pH variations after 24 hours of culture. **(A)** *Bacteroides intestinalis*, **(B)** *Bacteroides xylanisolvens*, **(C)** *Bacteroides thetaiotaomicron*, **(D)** *Bacteroides fragilis*, and **(E)** *Bacteroides uniformis*. Bacteria were cultured in a low-nutrient culture medium (LNCM) supplemented with different carbon sources, all at 0.5% (w/v): glucose or inulin, both used as positive controls (referred to as “glucose” or “inulin”); algae polysaccharide-enriched extracts (laminarin-enriched preparation from *Saccharina latissima*, ulvan-enriched preparation from *Ulva lactuca*, fucoidan-enriched preparation from *Undaria pinnatifida*, referred to as “laminarin,” “ulvan,” and “fucoidan,” respectively); and an RFO-enriched preparation from chickpeas, referred to as “RFO.” Purified commercial sources of raffinose and stachyose, referred to as “raffinose” and “stachyose,” respectively, were also included as model oligosaccharides found in RFO. A negative control was made without any added carbohydrates (referred to as “no carbohydrates”), corresponding to the basal LNCM. The top panels show changes in OD600 (ΔOD), reflecting bacterial growth, while the bottom panels show changes in pH (ΔpH), indicating fermentation activity. Negative pH variations indicate acidification of the medium due to fermentation. For each bacterial species and each condition, optical density (OD) and pH values were compared to the “No carbohydrates” condition using a two-sided Wilcoxon rank-sum test. Significance levels are indicated above bars (* $p < 0.05$; ** $p < 0.01$), and p-values were computed independently for each species across conditions. These results highlight the differential metabolic responses of bacterial species across phyla and their varying abilities to utilize specific dietary fibers.

The figures are presented on the following page.

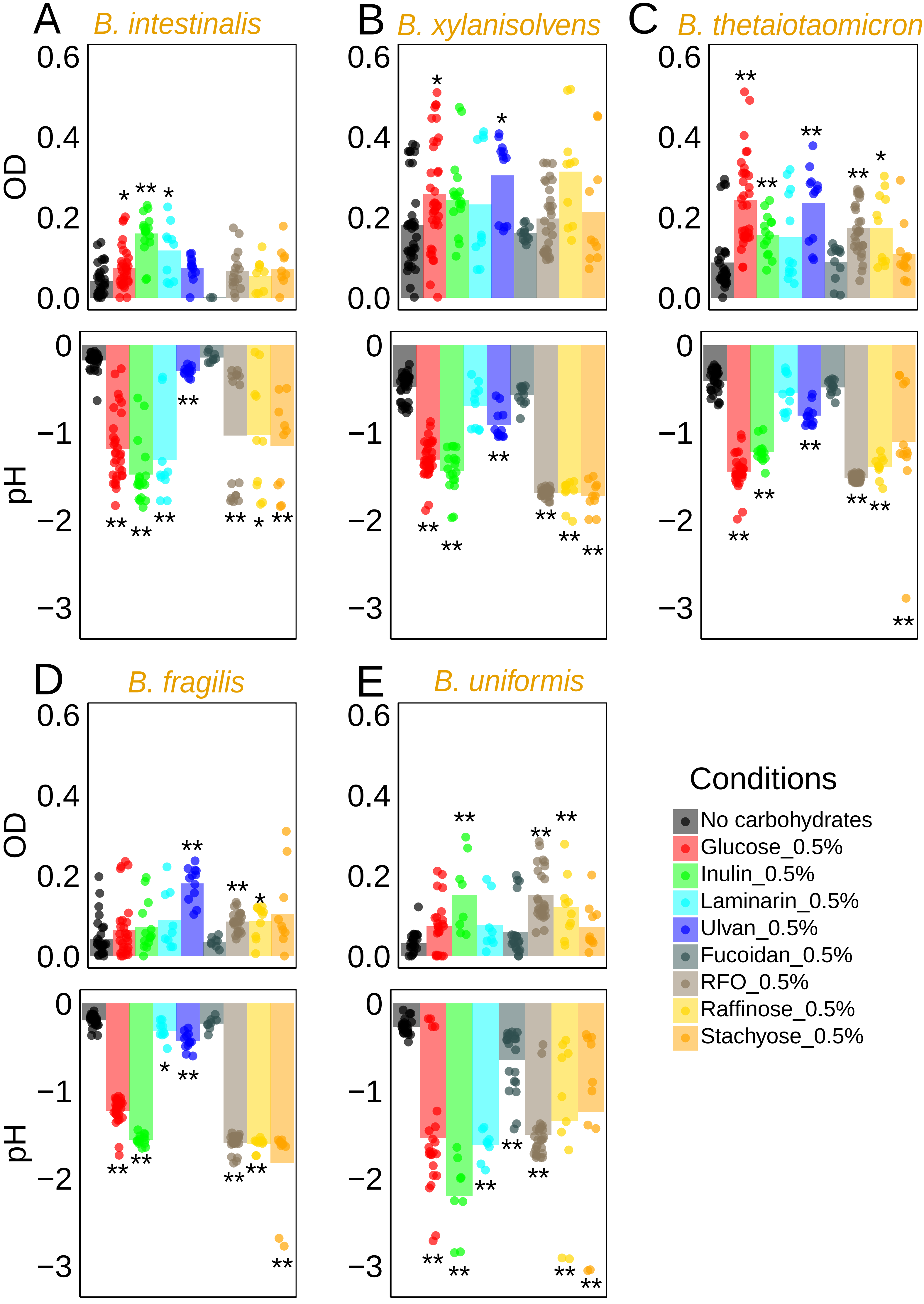
A
B. intestinalis
B
B. xylanisolvens
C
B. thetaiotaomicron
D
B. fragilis
E
B. uniformis
OD
pH
Conditions
No carbohydrates
Glucose_0.5%
Inulin_0.5%
Laminarin_0.5%
Ulvan_0.5%
Fucoidan_0.5%
RFO_0.5%
Raffinose_0.5%
Stachyose_0.5%

Supplementary Figure S2 : Growth and fermentation activity of intestinal commensal bacteria of the *Actinomycetota* phylum, reported individually, in the presence of fibers derived from algae and chickpeas.

Bacterial growth and fermentation activity were assessed by measuring optical density (OD600) and pH variations after 24 hours of culture. **(A)** *Bifidobacterium adolescentis* and **(B)** *Bifidobacterium catenulatum*. Bacteria were cultured in a low-nutrient medium (LNCM) supplemented with different carbon sources, all at 0.5% (w/v): glucose or inulin, both used as positive controls (referred to as "glucose" or "inulin"); algae polysaccharide-enriched extracts (laminarin-enriched preparation from *Saccharina latissima*, ulvan-enriched preparation from *Ulva lactuca*, fucoidan-enriched preparation from *Undaria pinnatifida*, referred to as "laminarin," "ulvan," and "fucoidan," respectively); and an RFO-enriched preparation from chickpeas, referred to as "RFO." Purified commercial sources of raffinose and stachyose, referred to as "raffinose" and "stachyose," respectively, were also included as model oligosaccharides found in RFO. A negative control was made without any added carbohydrates (referred to as "no carbohydrates"), corresponding to the basal LNCM. The top panels show changes in OD600 (ΔOD), reflecting bacterial growth, while the bottom panels show changes in pH (ΔpH), indicating fermentation activity. Negative pH variations indicate acidification of the medium due to fermentation. For each bacterial species and each condition, optical density (OD) and pH values were compared to the "No carbohydrates" condition using a two-sided Wilcoxon rank-sum test. Significance levels are indicated above bars (* $p < 0.05$; ** $p < 0.01$), and p-values were computed independently for each species across conditions. These results highlight the differential metabolic responses of bacterial species across phyla and their varying abilities to utilize specific dietary fibers.

The figures are presented on the following page.

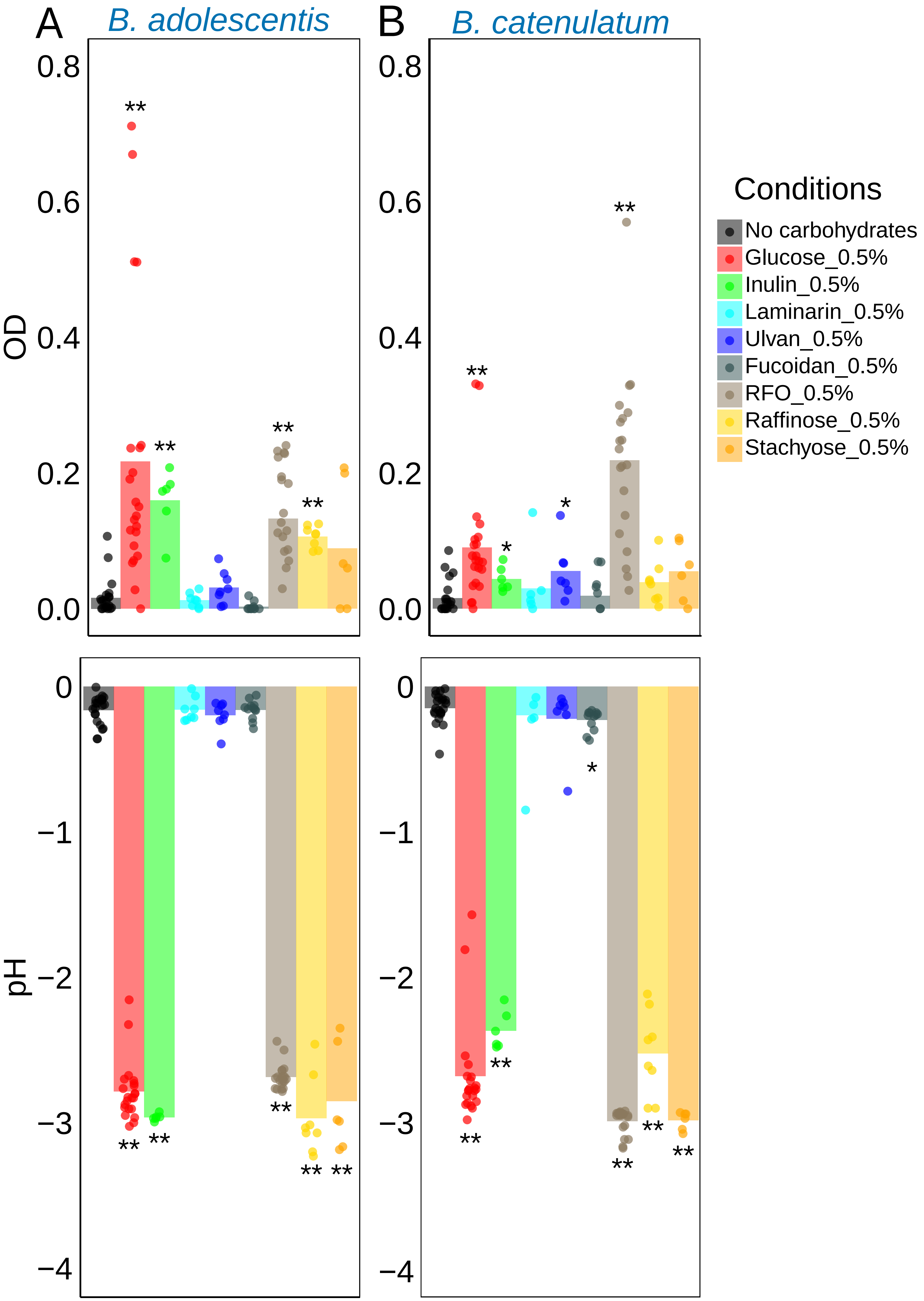
A
B. adolescentis
B
B. catenulatum
OD
pH
Conditions
No carbohydrates
Glucose_0.5%
Inulin_0.5%
Laminarin_0.5%
Ulvan_0.5%
Fucoidan_0.5%
RFO_0.5%
Raffinose_0.5%
Stachyose_0.5%

Supplementary Figure S3 : Growth and fermentation activity of intestinal commensal bacteria of the *Bacillota* phylum, reported individually, in the presence of fibers derived from algae and chickpeas.

Bacterial growth and fermentation activity were assessed by measuring optical density (OD600) and pH variations after 24 hours of culture. **(A)** *Faecalibacterium duncaniae*, **(B)** *Subdoligranulum variabile*, **(C)** *Butyricicoccus pullicaecorum*, **(D)** *Roseburia intestinalis*, **(E)** *Agathobacter rectalis*, **(F)** *Blautia hansenii*, **(G)** *Anaerostipes caccae*, and **(H)** *Anaerobutyricum hallii*. Bacteria were cultured in a low-nutrient medium (LNCM) supplemented with different carbon sources, all at 0.5% (w/v): glucose or inulin, both used as positive controls (referred to as "glucose" or "inulin"); algae polysaccharide-enriched extracts (laminarin-enriched preparation from *Saccharina latissima*, ulvan-enriched preparation from *Ulva lactuca*, fucoidan-enriched preparation from *Undaria pinnatifida*, referred to as "laminarin," "ulvan," and "fucoidan," respectively); and an RFO-enriched preparation from chickpeas, referred to as "RFO." Purified commercial sources of raffinose and stachyose, referred to as "raffinose" and "stachyose," respectively, were also included as model oligosaccharides found in RFO. A negative control was made without any added carbohydrates (referred to as "no carbohydrates"), corresponding to the basal LNCM. The top panels show changes in OD600 (ΔOD), reflecting bacterial growth, while the bottom panels show changes in pH (ΔpH), indicating fermentation activity. Negative pH variations indicate acidification of the medium due to fermentation. For each bacterial species and each condition, optical density (OD) and pH values were compared to the "No carbohydrates" condition using a two-sided Wilcoxon rank-sum test. Significance levels are indicated above bars (* $p < 0.05$; ** $p < 0.01$), and p-values were computed independently for each species across conditions. These results highlight the differential metabolic responses of bacterial species across phyla and their varying abilities to utilize specific dietary fibers.

The figures are presented on the following page.

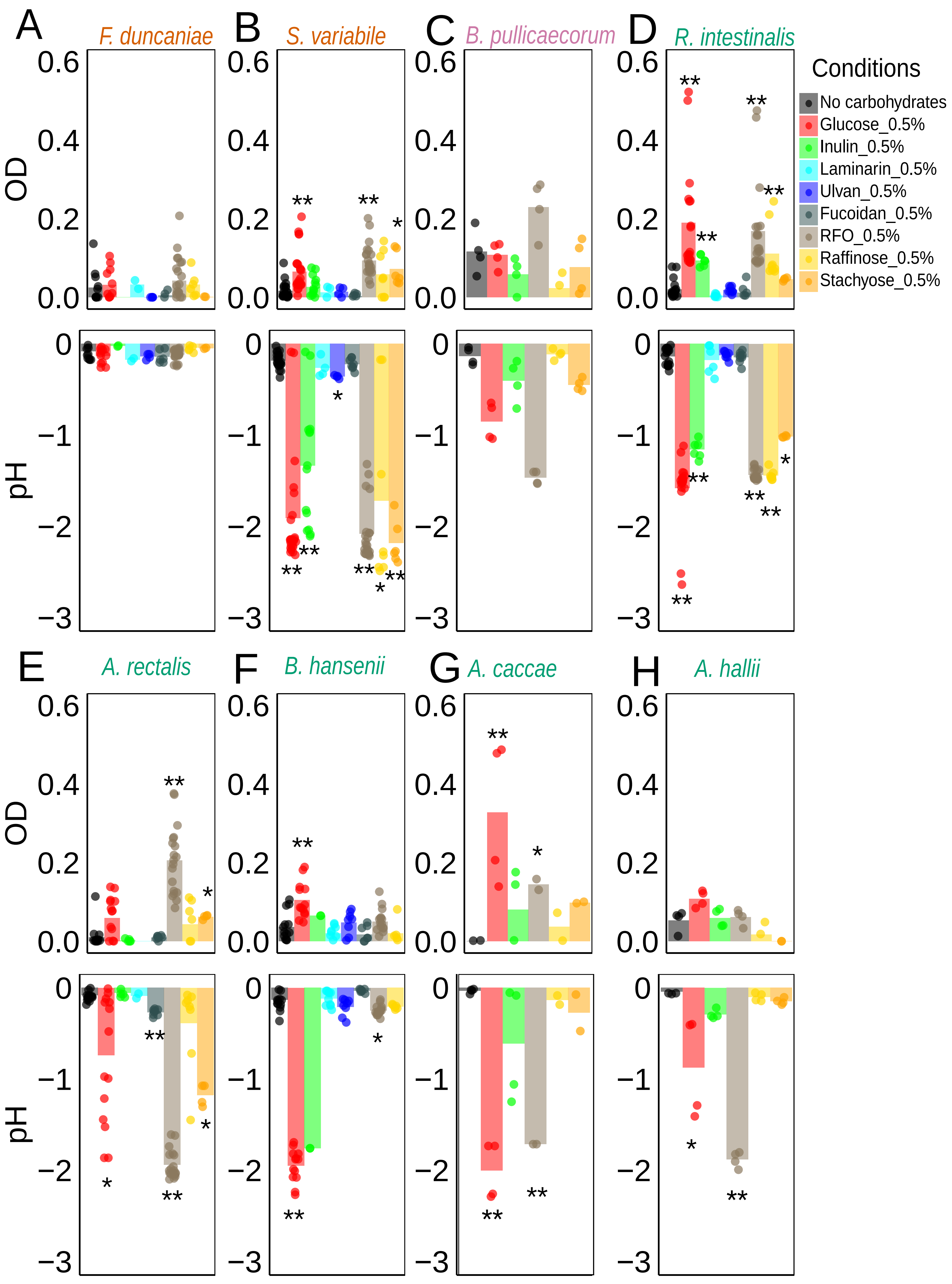

A
F. duncaniae
B
S. variabile
C
B. pullicaecorum
D
R. intestinalis
E
A. rectalis
F
B. hansenii
G
A. caccae
H
A. hallii
OD
pH
Conditions
No carbohydrates
Glucose_0.5%
Inulin_0.5%
Laminarin_0.5%
Ulvan_0.5%
Fucoidan_0.5%
RFO_0.5%
Raffinose_0.5%
Stachyose_0.5%

Supplementary Figure S4 : Growth and fermentation activity of *Bacteroidota* species in response to laminarin.

Bacterial growth and fermentation activity of *Bacteroidota* species (*Bacteroides intestinalis*, *Bacteroides thetaiotaomicron*, *Bacteroides fragilis*, *Bacteroides uniformis*, and *Bacteroides xylanisolvens*) were assessed in the presence of purified commercial laminarin (0.5% w/v). (A) Change in OD600 (ΔOD), representing bacterial growth. (B) pH variations (ΔpH), indicating fermentation activity. Negative values indicate acidification of the medium due to fermentation. For each species, values obtained under the laminarin condition were statistically compared to the no-carbohydrate control using a Wilcoxon rank-sum test. Statistical significance is indicated on the plots (*$p < 0.05$; **$p < 0.01$).

The figures are presented on the following page.

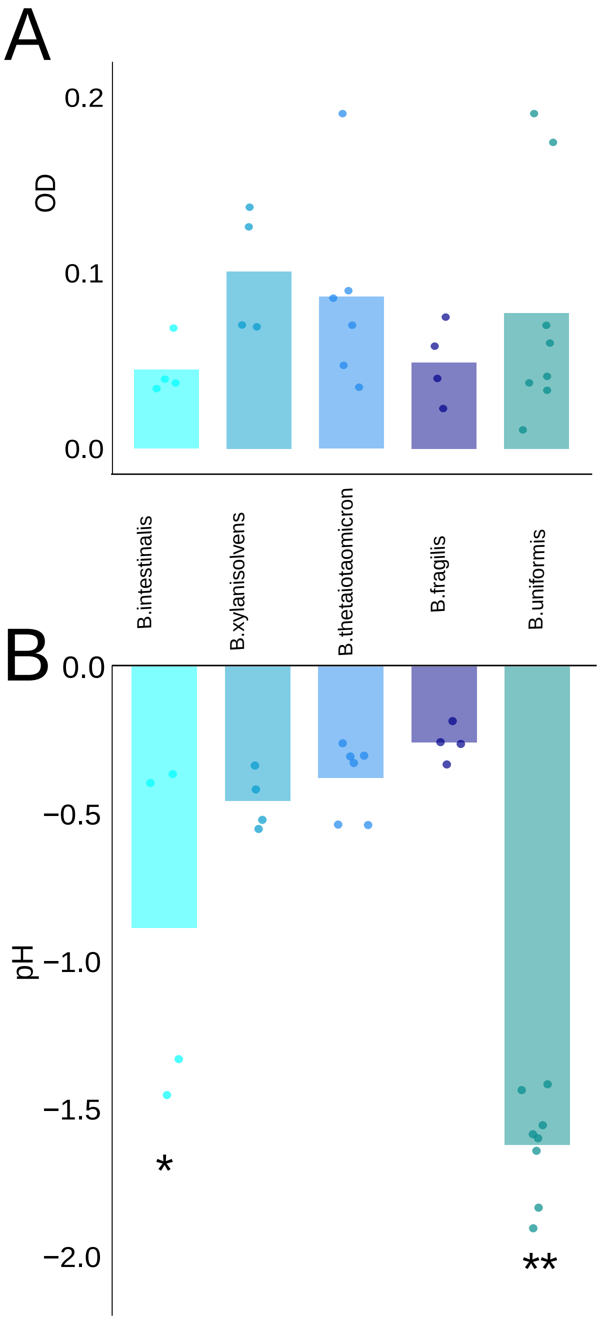

A
0.2
0.1
0.0
OD
B.intestinalis
B.xylanisolvens
B.thetaiotaomicron
B.fragilis
B.uniformis
B
0.0
−0.5
−1.0
−1.5
−2.0
pH
*
**

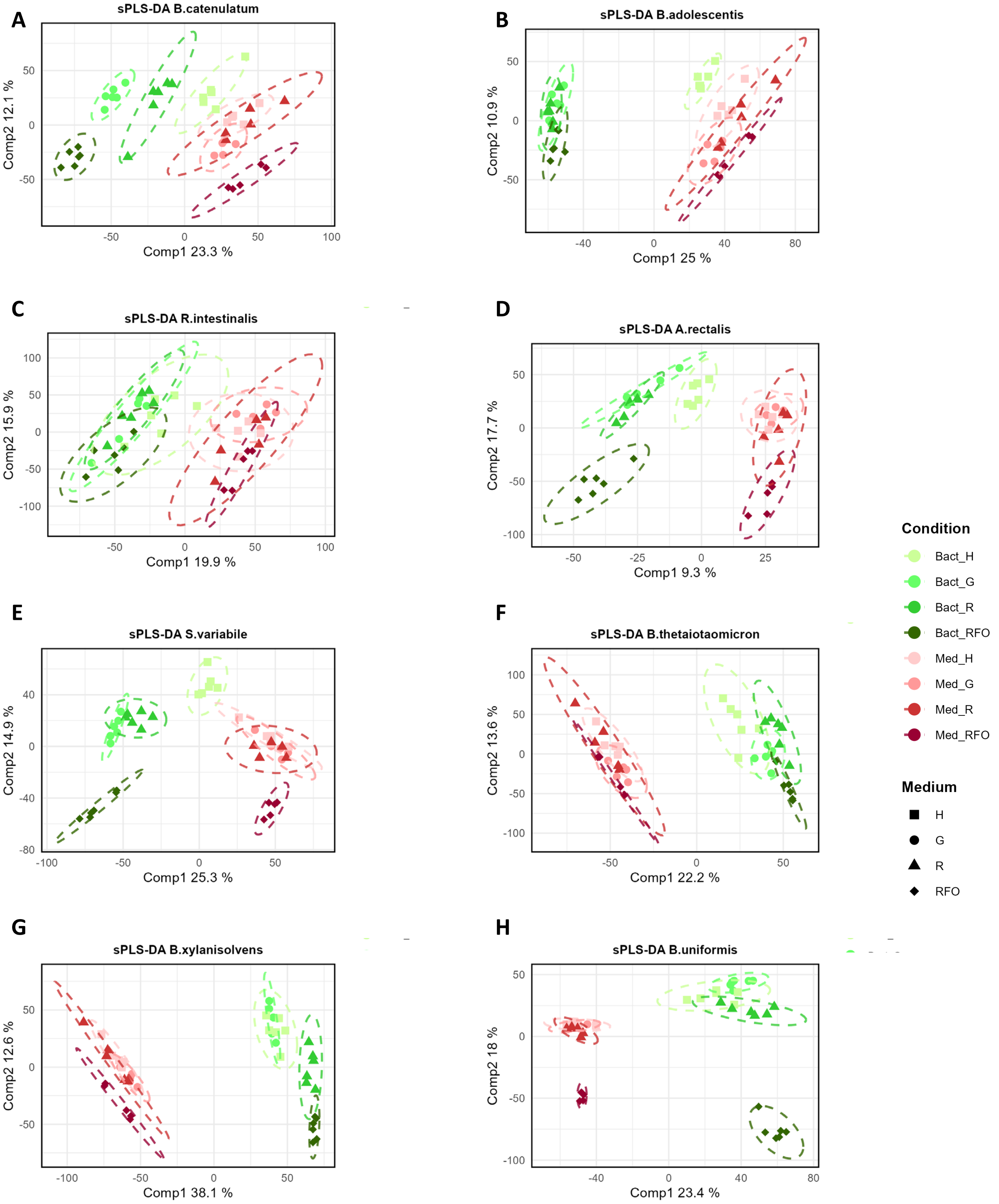

Supplementary Figure S5 : *Metabolomic profiles of commensal bacteria based on culture conditions and carbon sources.* *The metabolomic study was conducted on the supernatants of eight commensal bacteria from different bacterial phyla: Actinomycetota : Bifidobacterium catenulatum (A) Bifidobacterium adolescentis (B), Bacillota : Roseburia intestinalis (C), Agathobacter rectalis (D), Subdoligranulum variabile (E), and Bacteroidota : Bacteroides thetaiotaomicron (F), Bacteroides xylanisolvens (G), Bacteroides uniformis (H). These bacterial species were cultured in low-nutrient culture media (LNCM), each supplemented or not with different carbon sources : basal LNCM, is used as negative control and referred to as "H", glucose-supplemented LNCM is referred to as "G", purified raffinose-supplemented LNCM is referred to as "R" and RFO-supplemented LNCM is referred to as "RFO", added at 0.5% (w/v)]. Each condition was performed in six replicates, along with five replicates of the corresponding non-inoculated LNCM. Data was shown as scatter plots of the first two sparse partial least squares-discriminant analyses (sPLS-DA) components obtained for each bacteria.* Bact_H, Bact_G, Bact_R, Bact_RFO : media inoculated with bacteria; Med_H, Med_G, Med_R, Med_RFO : non-inoculated corresponding media. All ellipses were drawn assuming a multivariate t-distribution with a confidence level of 0.95.

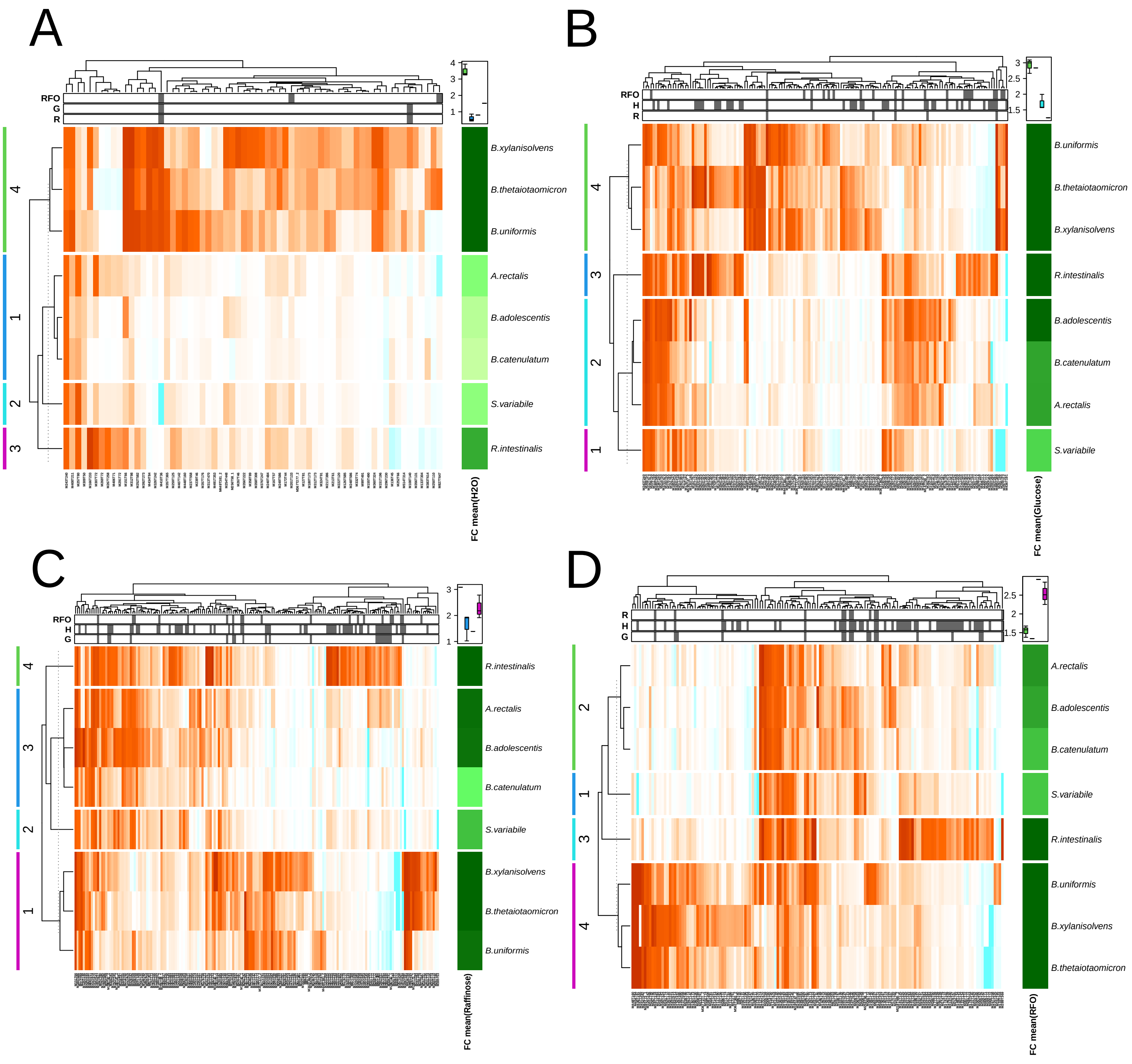

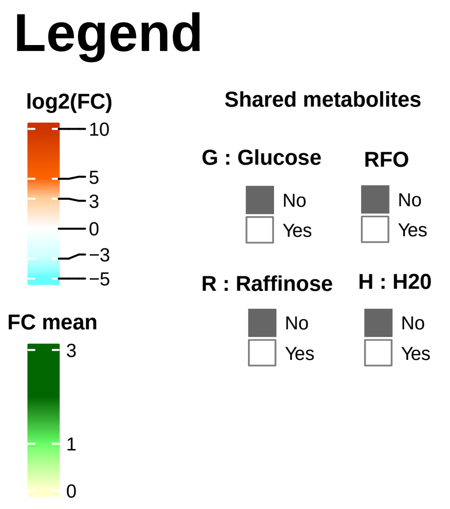

Supplementary Figure S6: Bacterial metabolites whose abundance is modified compared to the non-inoculated media. Heatmaps of differentially abundant metabolites after 24 hours of incubation of bacterial strains: (A) without any carbohydrate supplementation, in the presence of (B) 0.5% glucose, (C) 0.5% raffinose, and (D) 0.5% RFO-enriched preparation from chickpeas, referred to as " RFO". Metabolites annotated with public databases are shown. The bacterial species include: *Bacillota* (*Agathobacter rectalis, Roseburia intestinalis, Subdoligranulum variabile*), *Actinomycetota* (*Bifidobacterium adolescentis, Bifidobacterium catenulatum*), and *Bacteroidota* (*Bacteroides thetaiotaomicron, Bacteroides xylanisolvens, Bacteroides uniformis*). Metabolites that differed between the medium inoculated with each bacterial species and the corresponding non-inoculated medium were identified using a univariate non-parametric test (Wilcoxon test, Padj < 0.05). Metabolites with a fold change ≥ 10 in at least one bacteria per condition were included. The colored lines to the left of the heatmap identify clusters, with the associated dendrogram. The top heatmap annotation indicates whether metabolites are shared ("Yes") or not ("No") with the other culture media (H: basal LNCM; G: LNCM-Glucose; R: LNCM-Raffinose; RFO: LNCM-RFO). The single-column heatmap on the right represents the mean fold change for each row (i.e., per bacterium). The top boxplot summarizes the mean fold change for each of the clusters in the order of their appearance from top to bottom of the main heatmap.
