# Supplementary material for: Interactions between gut commensal bacteria and polysaccharides derived from algae and legumes: identification of metabolites produced and pathways involved
Source: Curr Res Microb Sci. 2026 Feb 10;10:100567. doi: 10.1016/j.crmicr.2026.100567 (PMC12924908; doi:10.1016/j.crmicr.2026.100567)
Supplement: Supplementary file 2 [file mmc2.pdf]

A

**Source**	*Ulva lactuca*	*Saccharina latissima*	*Undaria pinnatifida*	*Cicer arietinum*
**Sugar amount (% of extract)**	51%	33%	31%	28%

B

**Source**	**Poly/oligosaccharide**	**Molecular Weight (kDa)**	**Osidic composition (%)**
Ulva lactuca (algae)	Ulvan	830	49% rhamnose, 27% glucuronic acid, 10% iduronic acid, 10% glucose, 4% xylose
Saccharina latissima (algae)	Laminarin	5.4	100% glucose
Undaria pinnatifida (algae)	Fucoïdan	>100	35% fucose, 20% galactose, 1% mannose, 1% glucose
Cicer arietinum (pulses)	Raffinose family oligosaccharides (RFO)	<5	19% stachyose, 3% raffinose, <1% verbascose, sucrose detected

Supplementary Table S1: Characterization of poly/oligosaccharide-enriched extracts used in the study **(A)** Total sugar content of algae and chickpea extracts was determined according to Dubois *et al.* [1] **(B)** Carbohydrate monomers were analyzed using a high-performance anion-exchange chromatography system (HPAEC) with pulsed amperometric detection (PAD) and by gas chromatography with flame ionization detection (GC-FID), following the method of Kamerling *et al.* [2], as modified by Baussant *et al.* [3]. Standards (fucose, arabinose, rhamnose, glucose, galactose, mannose, xylose, ribose, glucuronic acid and iduronic acid) were run in parallel. The polysaccharides from *Ulva lactuca*, *Saccharina latissima*, and *Undaria pinnatifida* corresponded to ulvan, laminarin, and fucoidan, respectively, as indicated by their monomer profiles (presence of xylose, rhamnose, glucuronic acid, and iduronic acid for ulvan [4] ; glucose exclusively for laminarin [5], predominantly fucose for fucoidan [6]). The chickpea extracts were composed of the raffinose family oligosaccharides (RFO), and sucrose was also detected as expected [7] (data not shown).

[1] Dubois M, Gilles K, Hamilton JK, Rebers PA, Smith F. A colorimetric method for the determination of sugars. Nature 1951;168:167. https://doi.org/10.1038/168167a0.

[2] Kamerling JP, Gerwig GJ, Vliegenthart JF, Clamp JR. Characterization by gas-liquid chromatography-mass spectrometry and proton-magnetic-resonance spectroscopy of pertrimethylsilyl methyl glycosides obtained in the methanolysis of glycoproteins and glycopeptides. Biochem J 1975;151:491–5. https://doi.org/10.1042/bj1510491.

[3] Baussant T, Strecker G, Wieruszeski JM, Montreuil J, Michalski JC. Catabolism of glycoprotein glycans. Characterization of a lysosomal endo-N-acetyl-beta-D-glucosaminidase specific for glycans with a terminal chitobiose residue. Eur J Biochem 1986;159:381–5. https://doi.org/10.1111/j.1432-1033.1986.tb09879.x.

[4] Kidgell JT, Magnusson M, de Nys R, Glasson CRK. Ulvan: A systematic review of extraction, composition and function. Algal Research 2019;39:101422. https://doi.org/10.1016/j.algal.2019.101422.

[5] Liu Z, Xiong Y, Yi L, Dai R, Wang Y, Sun M, et al. Endo-β-1,3-glucanase digestion combined with the HPAEC-PAD-MS/MS analysis reveals the structural differences between two laminarins with different bioactivities. Carbohydr Polym 2018;194:339–49. https://doi.org/10.1016/j.carbpol.2018.04.044.

[6] Anjana K, Arunkumar K. Brown algae biomass for fucoxanthin, fucoidan and alginate; update review on structure, biosynthesis, biological activities and extraction valorisation. Int J Biol Macromol 2024;280:135632. https://doi.org/10.1016/j.ijbiomac.2024.135632.

[7] Elango D, Rajendran K, Van der Laan L, Sebastiar S, Raigne J, Thaiparambil NA, et al. Raffinose Family Oligosaccharides: Friend or Foe for Human and Plant Health? Front Plant Sci 2022;13:829118. https://doi.org/10.3389/fpls.2022.829118.
